# Axial Length Changes Following Surgical Intervention in Children With Primary Congenital Glaucoma

**DOI:** 10.3389/fopht.2021.747801

**Published:** 2021-11-01

**Authors:** Hind A. Al Dalgan, Ibrahim A. Al Obaida, Adi M. Al Owaifeer, Khabir Ahmad, Rizwan Malik

**Affiliations:** ^1^ Glaucoma Division, King Khaled Eye Specialist Hospital, Riyadh, Saudi Arabia; ^2^ Faculty of Ophthalmology, College of Medicine, Imam Abdulrahman bin Faisal University, Al-Khobar, Saudi Arabia; ^3^ Faculty of Ophthalmology, College of Medicine, King Faisal University, Al-Ahsa, Saudi Arabia; ^4^ Research Department, King Khaled Eye Specialist Hospital, Riyadh, Saudi Arabia; ^5^ Department of Ophthalmology and Vision Science, University of Alberta, Edmonton, AB, Canada

**Keywords:** childhood glaucoma, axial length (AL), intraocular pressure (IOP), surgery, primary congenital glaucoma (PCG)

## Abstract

**Background:**

Primary congenital glaucoma (PCG) is a challenging condition to diagnose, treat and effectively monitor. Serial assessment of intraocular pressure (IOP), optic disc cupping, refraction, and axial length (AxL) after surgery are useful to assess disease control. This study aimed to evaluate AxL changes in relation to IOP changes following glaucoma surgery in children with PCG.

**Methods:**

We retrospectively studied AxL changes in children with PCG undergoing surgery. Eyes of children aged ≤ 4 years that did not have prior ocular surgery and that underwent at least one glaucoma surgery during the course of follow-up between June 2014 and July 2018, were included. The effect of change in IOP on change in AxL was estimated using linear mixed effects models.

**Results:**

A total of 105 eyes (of 72 children) with PCG underwent glaucoma surgery representing 26.4% (105/397) eyes. The mean ± SD age of children at baseline was 3.53 ± 4.04 months. At baseline, the mean IOP and AxL were 26.63 ± 9.57 mmHg and 21.67 ± 1.82 mm, respectively. During the course of follow-up post-surgery, the IOP decreased by a mean of 7.25 ± 12.08 mmHg while the AxL increased by a mean of 0.70 ± 1.40 mm. A multivariable mixed effects linear regression revealed that change in AxL was significantly associated with change in IOP (p=0.030) and time since first surgery (p<0.001). A substantial reduction in IOP (≥35 mmHg) was needed at 3 months post-surgery, for AxL to regress.

**Conclusion:**

In children with PCG who undergo glaucoma surgery, change in IOP significantly influences change in AxL. For AxL to regress, a substantial reduction in IOP is needed post-surgery.

## Introduction

Congenital glaucoma is one of the major causes of blindness during childhood (1). It is characterized by elevated IOP, optic nerve damage and globe enlargement manifested as buphthalmos, increased corneal diameter and increased axial length (AxL) (2).

Primary congenital glaucoma (PCG) is the most common form of congenital glaucoma. It is a developmental eye disorder due to an isolated maldevelopment of anterior chamber angle, the major site of aqueous humor drainage, without an identifiable ocular or systemic mechanism. Eighty-percent of children present before 1 year of age (3).

Surgery is the primary therapeutic modality in PCG, while medications have a supportive role (4). The surgical options include goniotomy, trabeculotomy, trabeculectomy, with comparable success rates (5), combined trabeculectomy-trabeculotomy (6) and non-penetrating deep sclerectomy (NPDS) (7). Glaucoma drainage devices (GDD) and cyclodestructive procedures are reserved for refractory cases (8).

PCG is a challenging condition to diagnose, to manage and to monitor for disease progression. Serial follow-up is necessary after any surgical intervention to assess the disease control. For that purpose, IOP, optic disc cupping, refraction, and AxL are useful modalities for monitoring in children (8, 9).

Axial myopia is an important clinical finding in PCG and is the result of high IOP on the thin elastic sclera of children with congenital glaucoma (9). In healthy children, AxL increases with age with steepest growth at 10 months till it is almost stabilized at 3 years of age (10). In younger children with glaucoma, IOP influences the degree of axial length growth (11). Further, evidence suggests that surgical or medical IOP reduction may reduce AxL (8, 12), so it is a valuable tool in assessing response to surgical intervention.

For axial length to serve as a useful indicator of adequate IOP reduction following surgery, the relationship between AxL change and IOP change needs to be quantified. Few studies in the literature demonstrated AxL change in relation to IOP change after glaucoma surgery in childhood glaucoma. One study showed a good correlation between post-operative IOP and post-operative AxL growth after trabeculotomy (12, 13) and goniotomy (14) while another study showed no correlation after trabeculectomy (15).

We recently reported findings of one of the largest studies (n=397 phakic eyes of 208 children) to assess the relationship of axial length, and age in children with primary congenital glaucoma (16), but that study did not quantify the effect of surgery on AxL. This present article reports our findings regarding the axial length changes in relation to IOP changes specifically following glaucoma surgery in these children.

## Materials and Methods

### Study Design and Setting

We retrospectively analyzed data of children with PCG who had not undergone previous surgery and underwent at least one glaucoma surgery between June 2014 and July 2018 at a tertiary eye hospital in the Middle-East since the initiation of the electronic patient record system (TrakCare, Intersystems, Cambridge, MA). The records of patients with a diagnosis of ‘pediatric glaucoma’ (by hospital code) were reviewed. Ethics approval was obtained from the Institutional review board, IRB (IRB no 1837-R) and the study adhered to the tenets of the Declaration of Helsinki.

### Inclusion and Exclusion Criteria

Eyes of children aged ≤ 4 years that did not have prior ocular surgery, underwent at least one glaucoma surgery during the course of follow- up at our institution and completed 3-month post-operative follow-up, were included. We confined our study to this age group because AxL is not routinely measured at our institution beyond this age, as it reaches a plateau after 4 years of age (17).

For unilateral disease only the affected eye was included. For bilateral disease both eyes were included. Our analysis accounted for inclusion of both eyes from each child (see *Data Analysis* below) to account for inter-eye correlation.

Eyes with, secondary glaucoma, pseudophakic or aphakic, blind or phthisical eyes and eyes that did not have any AxL data during the study period were excluded.

### Data Collection and Diagnostic Criteria

PCG was defined as elevated IOP (> 21 mmHg) associated with enlarged corneal diameter (> 11 mm in newborn or > 12 mm for older children), corneal haze, increased cup to disc ratio (>0.4) or asymmetric cupping of optic nerve head between the two eyes in the absence of associated ocular or systemic anomalies.

The following data were collected: age, gender, IOP, ocular AxL, and number of anti-glaucoma medications, number of glaucoma surgeries during follow up, and time since glaucoma surgery.

Preoperative and follow-up examinations, including IOP and AxL, were done after oral Chloral Hydrate sedation or general anesthesia (GA) just before intubation, with sevoflurane as the induction agent. Slit lamp biomicroscopic examination of the anterior segment, corneal diameter measurement, fundoscopy with special attention to the optic disc appearance, IOP measurement, central corneal thickness and sonographic measurement of AxL were performed. Refraction was done at each visit, typically at 3-4 monthly intervals. The decision for surgery was based on the pre-operative sedation IOP.

IOP was measured using Tonopen AVIA (TPA, Reichert Inc. NY, USA) after applying topical anaesthesia (0.4% benoxinate). The Perkins tonometer was not used as we have found it often to give erroneous readings in scarred or oedematous corneas (18).

AxL measurements were taken by an experienced ophthalmic technician utilizing a 10 MHz A-scan machine (Cinescan, Quantel Medical, Cournon d’Auvergne, France) using contact biometry. After application of a topical anesthetic agent the probe was placed gently on the child’s cornea. Characteristic waves representing cornea, lens, retina, and sclera were observed. Prior to taking a reading the ultrasound waves were evaluated and in cases of a noisy signal, the reading was repeated. The AxL was defined as the distance from the wave representing the anterior corneal surface to the wave representing the retina at the macula. In each patient, the machine recorded five accurate axial length measurements and gave an average reading of all measurements.

### Surgery and Post-Operative Care

Glaucoma surgeries performed for PCG include trabeculectomy, NPDS, GDD, combined trabeculectomy-trabeculotomy and trans-scleral cyclophotocoagulation as primary or secondary glaucoma procedures. Given the poor outcome of angle surgery in Middle-Eastern CYP1B1 genotype of glaucoma (19) and the high success of NPDS in our population (7), most children underwent NPDS as the primary procedure. Our usual algorithm for surgical management was NPDS, followed by a second NPDS or a trabeculectomy. In cases of failure, a needling was often tried before considering a glaucoma drainage device.

The surgical techniques have been mentioned elsewhere (3, 7, 20–22). The postoperative medications included topical Prednisolone 1% tapered over 4-8 weeks and Moxifloxacin every 6 hours for 1-2 weeks, Atropine 0.5% every 12 hours or Cyclopentolate 0.5% every 8 hours for 2 weeks if needed.

Routine post-operative exams were conducted at the first day post-operatively, 1 month then every two to six months depending on the age of the child, IOP and severity of the disease.

### Outcome Measures and Definitions

The *change in AxL* following glaucoma surgery was computed as the algebraic difference of AxL after surgery minus the AxL prior to the surgery, so that a negative change indicated a regression of AxL after surgery. Similarly, the change in IOP was computed as post-surgery minus pre-surgery.

### Data Analysis

Data were entered using Microsoft Excel 2010 (Microsoft Corporation, Redmond, Washington) and analyzed using STATA 16.1 (StataCorp LLC, College Station, TX, USA). Frequencies, percentages and means with standard deviation were computed to describe the study population.

An exploratory data analysis was undertaken to visualize trends in the data. A scatterplot of change in AxL (mm) versus change in IOP (mmHg) over the entire follow up was plotted. A locally weighted smoothing (LOWESS) curve and a linear fit were applied to the data to visualize the relationship of change in AxL with change in IOP (mmHg).

Subsequently, the effect of change in IOP on change in AxL was estimated using linear mixed effects models, for all included eyes. The following independent variables were considered for the crude analysis: age and AxL at baseline, gender, the initial glaucoma surgery, time since surgery, and change in IOP, using child’s ID and eye as random intercepts to account for multiple observations per patient and per eye.

A multivariable age- and sex-adjusted mixed effect model was developed. The −2 Log likelihood and Akaike’s information criterion (AIC) values were used to identify the model with the best fit. The lower the −2 Log Likelihood and AIC values, the better the model.

STATA’s margins command was used to compute predicted change in AxL with time after surgery, for different levels of IOP change. Subsequently, the *marginsplot* command within STATA was used to graph predicted AxL changes from fitted model for 3, 6, 12 and 18 months after surgery.

## Results

We identified 397 phakic eyes (of 208 children) with PCG during the study period. Of these 138 eyes (of 93 children) underwent surgery during the course of follow-up and formed the eligible sample for the current study. The other eyes had received surgery prior to the period of the study. A total of 105 eyes (of 72 children), for which Tonopen^®^ IOP measurements were available, were included in this analysis. The mean ± SD age of children at the baseline visit was 3.53± 4.04 months with a mean ± SD AxL of 21.67 ± 1.82 mm, **Table 1**.

**Table 1:** Baseline characteristics of study population (105 eyes of 93 children).

Parameter	Value
Age (months), mean± SD	3.53 ± 4.04
Gender (number), boys: girls	31: 41
Number of anti-glaucoma medications (AGM), mean ± SD	2.31 ± 1.51
IOP (mmHg), mean ± SD	26.63 ± 9.57
Axial length (mm), mean ± SD	21.67 ± 1.82

SD, standard deviation; IOP, intraocular pressure.

As far as the initial glaucoma surgery, eighty-three eyes (79.0%) underwent NPDS, 10 (9.6%) underwent trabeculectomy, 7 (6.7%) underwent trabeculotomy and trabeculectomy (T & T), 2 (1.9%) had trans-scleral cyclophotocoagulation and 3 (2.9%) underwent other surgeries.

**Figure 1:**
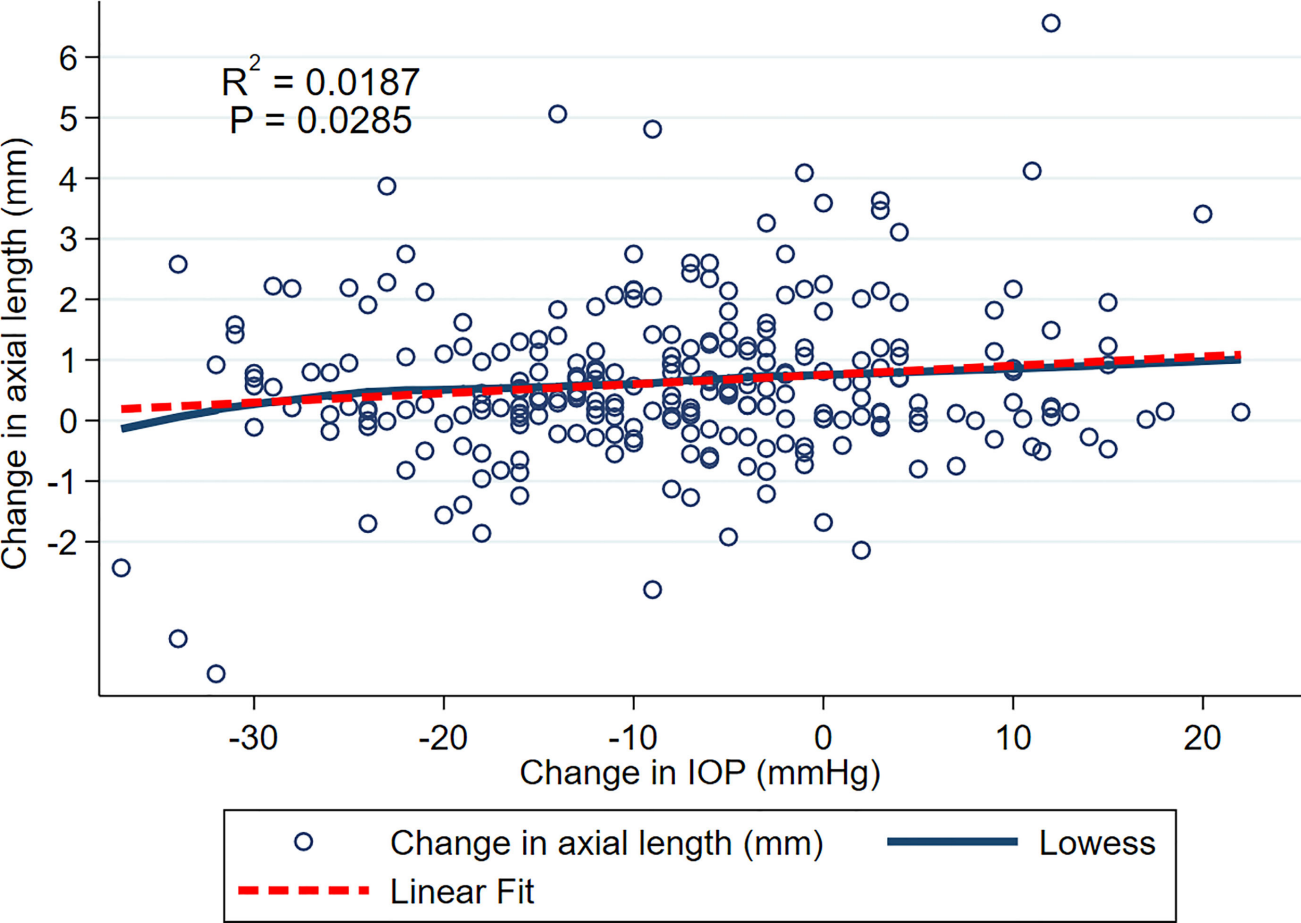
Scatterplot of change in AxL and change in IOP over the entire follow up. LOWESS and linear fits to the data are shown.

**Figure 1** shows relationship between change in AxL (mm) and change in IOP (mmHg) across all included eyes and all time points, with both LOWESS and linear fits to the data. Although there was a general trend for change in AxL to increase with change in IOP, the linear relationship was weak (R^2 =^ 0.0187, p<0.028). Across all measurements and time points, the mean increase in AxL was 0.70 ± 1.40 mm and mean reduction in IOP was 7.25 ± 12.08 mmHg, with 39% of eyes achieving an IOP reduction of greater than 10 mmHg.

**Figure 2:**
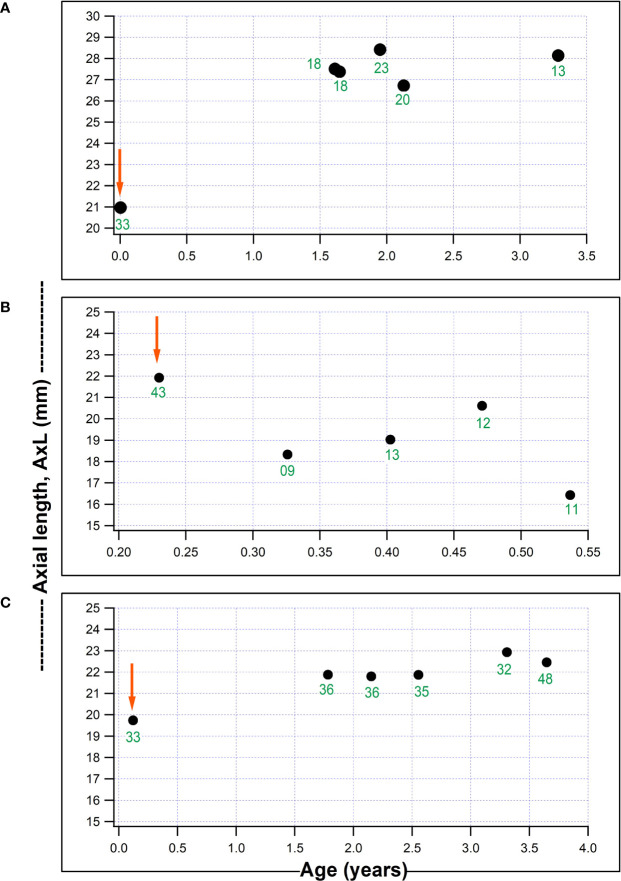
Three individual plots of axial length (AxL) change with age in our series. **(A)** Child who underwent non-penetrating deep sclerectomy, NPDS in the right eye aged 18 days old and AxL continued to increase despite a reduction in IOP; **(B)** Child who underwent NPDS in the right eye aged 2.8 months and had a AxL reduction post-operatively which was associated with a marked IOP reduction; **(C)** Child aged 44 days, with a failed NPDS in the left eye, without a reduction in post-operative IOP and AxL which remained fairly stable post-surgery (red arrows represents timing of surgical intervention and numbers in green font indicate IOP (mmHg) at each visit).

**Figure 2** shows plots for individual children for AxL against age (years), with a child who had a substantial IOP reduction from surgery but with a continued increase in AxL post-operatively (**Figure 2A**); one child with IOP reduction and AxL regression after surgery (**Figure 2B**) and one child with failed surgery with continued AxL increase after surgery (**Figure 2C**). Whilst, in some children AxL reduced after IOP reduction, a common pattern was for AxL to continue increasing despite IOP reduction.

A crude mixed effects linear regression analysis revealed that AxL tended to significantly increase with change in IOP (p=0.027) and time since the surgery (p<0.001) and be inversely related to the age at surgery (p=0.037) **Table 1**. The baseline age- and sex adjusted- multivariable mixed effects linear regression analysis model that was developed is shown in **Table 2**. This Model showed that the change in AxL was significantly influenced by change in IOP (p=0.030) and follow-up time (p<0.001). The type of initial glaucoma surgery (DS) had no significant influence on the change in AxL (p=0.841).

**Table 2:** Linear mixed effect model of the relationship between change in axial length (AxL) and change in IOP – Crude analysis.

Change in axial length, AxL (mm)	Crude analysis			
	Coeff.	LCL	UCL	P
Change in IOP (mmHg)	0.02	0.002	0.03	0.027
Time since first surgery (months)	0.12	0.09	0.14	<0.001
Initial glaucoma surgery (NPDS)	-0.02	-0.41	0.36	0.907
Baseline AxL (mm)	-0.08	-0.18	0.02	0.115
Baseline age (months)	-0.04	-0.08	0.002	0.037
Sex (female)	-0.16	-0.51	0.18	0.352

IOP, intraocular pressure; LCL and UCL indicate the lower and upper 95% confidence limits, respectively.

**Figure 3:**
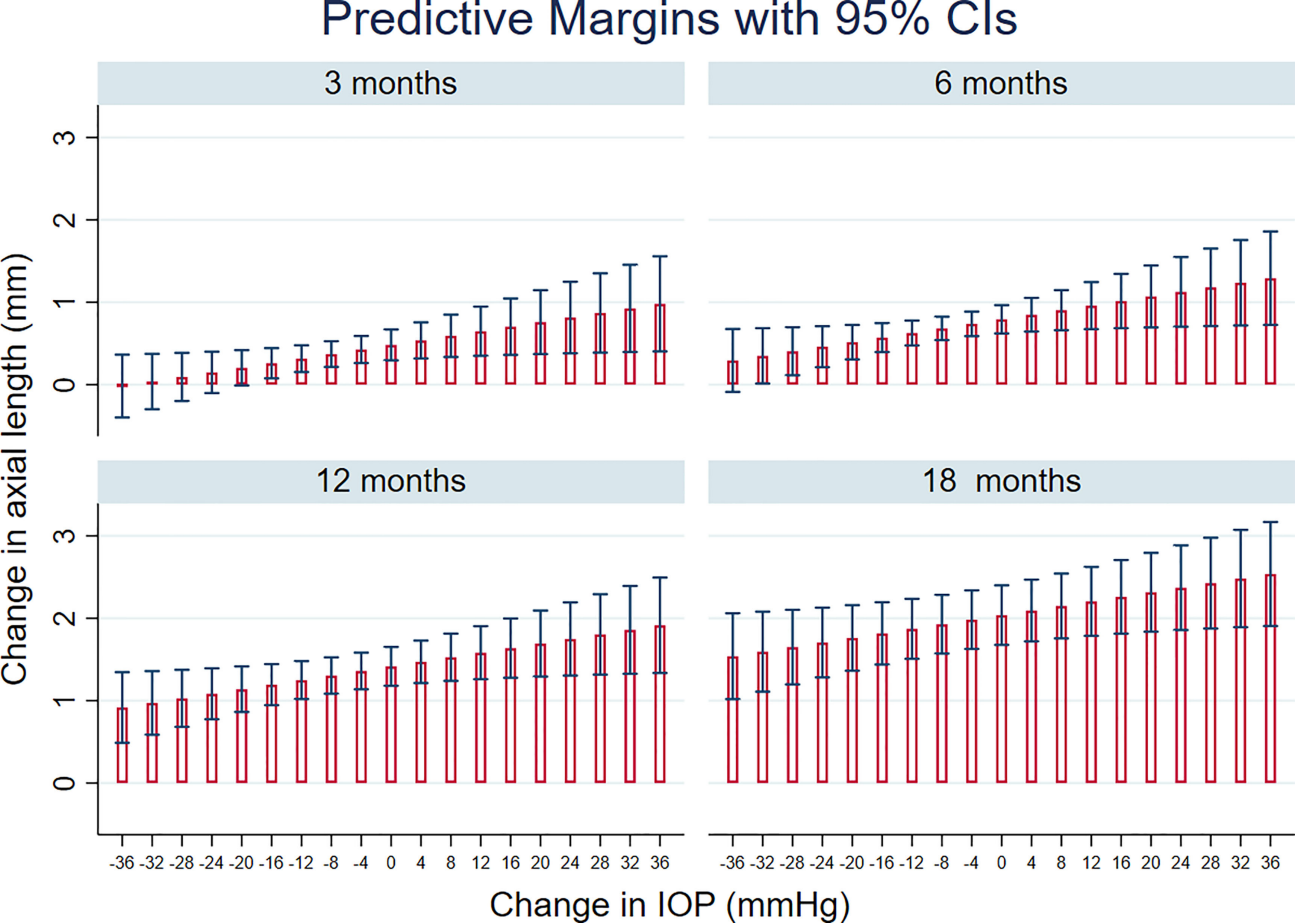
Linear mixed models-based plot of predicted change in axial length, AxL with time after surgery, for different levels of IOP change, based on the final multivariable regression model (see also **Table 3**).

**Figure 3** shows prediction from this model, in terms of the change in AxL for different levels of IOP and time after surgery. This predicted that, 12 months after surgery, mean increase in AxL of 1.41 mm (95% CI:1.18, 1.65) would be expected if there was no change in IOP after surgery; a mean increase in AxL of 1.36 mm (95% CI: 1.14, 1.58) would be expected for an IOP reduction of 4 mmHg, an increase of AxL of 1.30 mm (95% CI: 1.08, 1.52) for a reduction of IOP of 8 mmHg and a change of AxL of 1.25 mm (95% CI: 1.02, 1.48) for a reduction of IOP of 12 mmHg. A substantial reduction of IOP (≥ 35 mmHg) was needed, even at 3 months post-surgery, for AxL to regress (negative change).

## Discussion

Assessment of glaucomatous disease progression is challenging in young children, as perimetry is not possible in this age group (23). The measurement of IOP, the use of general anesthesia and corneal scarring can effect IOP reading in such children (3). In addition, IOP variability across different instruments and different times so serves only as a short-term parameter for the disease control (14, 24). Optic disc visualization can be hindered by corneal haze. Ocular AxL measurement has an important role in the assessment of eyes with PCG, especially in eyes with corneal opacity where assessment of optic disc progression may be difficult. AxL measurement can be performed under sedation or anesthesia and is a valuable tool in the diagnosis and follow-up, as a long-term parameter for disease control in children with congenital glaucoma (11, 14).

In this study we examined the relationship between IOP change and axial length change after glaucoma surgery in eyes with congenital glaucoma. The hypothesis was that the AxL reduction would be related to the degree of IOP reduction. However, the relationship was found to be weak in our exploratory analysis, with IOP change explaining less than 2% of the variance in AxL change (**Figure 1**). There are several possible explanations for this. Firstly, this may represent an actual dissociation between IOP and AxL. Secondly, AxL measurements and IOP measurements themselves are variable (25) and this variability is likely to contribute to the lack of correlation. Thirdly, it seems that the relationship between AxL and IOP is complex and dependent on a number of factors including baseline age and AxL, time since the surgery and the number of glaucoma surgeries during follow-up, as well as the degree of IOP reduction.

Some other investigators have also found such a weak relationship between AxL and IOP (13, 15). Cronemberger et al., reported ﻿no statistically significant correlation between IOP and other parameters including AxL in 32 eyes with PCG after being treated surgically (r = 0.24, corresponding to approximate R^2^ = 0.09). Pre-operatively, the mean IOP was 15.69 ± 5.31 mm Hg and the mean AxL was 24.57 ± 2.71 mm. At final follow-up, corresponding parameters were 6.16 ± 2.42 mm Hg and 25.37 ± 2.66 mm respectively (13). Matuszewska et al. also reported no correlation between IOP and AxL after trabeculectomy done for 36 eyes with PCG (15).

Contrary to our findings, Kiefer et al., studied the correlation of IOP and AxL change after trabeculotomy (TO) and goniotomy (GO) in 37 and 26 congenital glaucoma eyes respectively and showed a ﻿significant linear correlation (r=0.658, corresponding to approximate R^2^ = 0.43) (14). Alsheikheh et al., also reported a significant linear relationship (﻿r=0.48, corresponding to approximate R^2^ = 0.18) (25). The longer pre-operative AxL (22.3 ± 2.6 mm in TO, 22.5 ± 1.1 mm for GO in *Kiefer’s* and 22.6 ± 1.8 in *Alsheikheh*’s vs 21.73 ± 1.82 mm in this study) and the lower final mean IOP (﻿17.5 ± 5.8 mmHg for TO and 17.4 ± 10.2 mmHg for GO ﻿in *Kiefer’s*, 15.0 ± 3.9 mmHg in *Alsheikheh*’s vs 18.5 ± 7.2 mmHg in this study) may help explain the stronger correlation in the previous two studies. In adults with glaucoma, IOP change and AxL change has been found to be related. Usui et al., found a positive correlation between the reductions in IOP (from ﻿26.6 ± 7.6 mmHg preoperatively to 7.2 ± 2.6 mmHg postoperatively) and the shortening of AxL (from ﻿﻿25.12 ± 1.42 mm preoperatively to ﻿24.86 ± 1.43 mm postoperatively) in eyes that underwent trabeculectomy in adults (﻿R^2^ = 0.41) (26).

On average, a substantial reduction of IOP (≥ 35 mmHg) was needed, even at 3 months post-surgery, for AxL to regress (negative change) (**Figure 3**), after which there was a tendency for AxL to increase gradually. Post-operatively, choroidal thickening might contribute to the lower measured AxL in addition to the mechanical retraction of the globe after IOP reduction (26). ﻿*Németh* and *Horóczi* found that ocular wall thickness and volume increased and AxL decreased after trabeculectomy (27). Chakraborty et al., also observed a negative correlation between diurnal variations in AxL and choroidal thickness (28). At an individual level, we found different patterns of AxL change in relation to IOP change: a common pattern was for IOP to reduce but AxL to keep increasing after surgery (**Figure 2**). Physiological axial growth of the eye with age (10, 11) and possibly resolution of choroidal thickening might explain this AxL increase. Reduction of AxL following IOP reduction is uncommon (**Figure 2**). This is a finding consistent with other reports: Law et al. found out of 12 eyes undergoing surgery, only 2 showed a decrease in AxL (8). AxL regression may be more likely to occur in children with a longer eye and who have a marked IOP reduction post-operatively (**Table 2**). AxL change was found to be negatively correlated with age (**Table 3**), This is likely to be related to the elastic fiber content of the sclera in younger children (29).

**Table 3:** Linear mixed effect model of the relationship between change in axial length (AxL) and change in IOP – Multivariable analysis.

Change in axial length, AxL (mm)	Multivariable analysis*			
	Coeff.	LCL	UCL	P
Change in IOP (mmHg)	0.01	0.001	0.03	0.030
Baseline AxL (mm)	-0.07	-0.18	0.05	0.248
Initial glaucoma surgery (NPDS)	0.03	-0.29	0.36	0.841
Time since surgery (months)	0.10	0.08	0.13	<0.001
−2 Log likelihood	794.4923			
AIC	812.4923			

*Age and sex-adjusted

IOP, intraocular pressure; LCL and UCL indicate lower and upper 95% confidence limits respectively;

AIC, Akaike information criterion; (the lower the AIC, the better the fit of the model).

Limitations of our study include a retrospective design. The use of A-scan, which was used to measure AxL in this study, can falsely shorten the measured AxL by indenting the cornea (30). It may be this indentation is greater in eyes with lower IOP, leading to a dependence of AxL on IOP. As discussed above, IOP and AxL measurements are also subject to intra-individual, test-retest and inter-devices variability. Some of this variability can be reduced by taking an average of several measurements in a prospectively-designed study.

One further limitation is that there was no study-specific standardized protocol for measurement of IOP, either in terms of sedation/anaesthesia or the type of tonometer used. Some children had their IOP measured before intubation during GA, and other children had their IOP measured during sedation in the outpatient clinic. The results of one randomized trial of Sevoflurane and Ketamine suggested that the former may lower IOP (31), in which case it may be unfair to use measurements under Sevoflurane in our study. However, another study showed Sevoflurane not to significantly affect IOP measurements (32), this is consistent with our experience. Aside from anaesthetic agent, the type of tonometer affects the measured IOP and IOP measurement is dependent on the type of tonometer (33, 34). The present study ideally needs to be repeated in prospective manner with an *a priori* protocol for IOP measurement for all children.

One of the factors that may have contributed to IOP reduction post-operatively is change in corneal clarity and thickness post-surgery. As we did not study corneal thickness in a longitudinal manner, this is a potential confounder in our study.

We did not include a control group in the current study for several reasons. Firstly, the compliance of sclera in normal eyes and AxL change is likely to be different in healthy eyes, compared to eyes with PCG in which the sclera tends to be thinner (35). Secondly, there were no healthy children at our tertiary facility with longitudinal AxL and IOP measurements, such children would need to be recruited in a prospective study. Thirdly, there are ethical issues for recruiting such children, who do not need longitudinal measurements as part of their clinical care. We have reported AxL changes previously in another control group (children with PCG who did not have surgery) and that group serves as a useful control measure of AxL change.

Angle surgery alone is considered to carry a poor outcome in Middle-Eastern congenital glaucoma (19). Although NPDS has been reported to carry a good success in eyes with PCG (7), nearly half of patients actually had microperforation during the surgery in Al-Obeidan et al’s study. It is possible that angle surgery combined with filtration surgery may give the most optimal IOP-lowering in these eyes (36). In our personal experience, it seems that NPDS, which can be easily combined with trabeculotomy (37) offers a good surgical option, but much work is needed on establishing the ideal procedure for these eyes. Most of the surgeries performed in our sample were NPDS and it may be argued that the findings are not applicable to other populations. However, as the aim of all the surgeries is to reduce IOP, we expect the main findings of this study i.e. the influence of IOP reduction on AxL are still applicable.

A useful adjust to the findings of the study would have been to study the effect of surgical intervention on other parameters which would have been altered by IOP reduction, including corneal diameter, corneal clarity and refractive error. However, measurement of corneal clarity is subjective and refraction is often not possible in eyes with corneal scarring or opacity, thereby making axial length one of the most reproducible parameters in children with PCG.

In conclusion, there appears to be a weak linear association of IOP change and AxL change in children with PCG who undergo glaucoma surgery. An AxL reduction (3 months following surgery) is more likely in children who have a substantial IOP reduction from surgery.

## Data Availability Statement

The raw data supporting the conclusions of this article will be made available by the authors, without undue reservation.

## Ethics Statement

The studies involving human participants were reviewed and approved by King Khaled Eye Specialist Hospital IRB. Written informed consent from the participants’ legal guardian/next of kin was not required to participate in this study in accordance with the national legislation and the institutional requirements.

## Author Contributions

HD: conceptualization, project administration, data curation, investigation, writing (initial draft). AO: data curation, investigation, writing (editing and review). IO: data curation, investigation, writing (editing and review). KA: formal analysis, methodology, visualisation, writing, revisions (editing and review). RM: Conceptualisation, Supervision, methodology, visualisation, writing (editing and review). All authors contributed to the article and approved the submitted version.

## Conflict of Interest

The authors declare that the research was conducted in the absence of any commercial or financial relationships that could be construed as a potential conflict of interest.

## Publisher’s Note

All claims expressed in this article are solely those of the authors and do not necessarily represent those of their affiliated organizations, or those of the publisher, the editors and the reviewers. Any product that may be evaluated in this article, or claim that may be made by its manufacturer, is not guaranteed or endorsed by the publisher.
